# Monkeying Around: Using Non-human Primate Models to Study NK Cell Biology in HIV Infections

**DOI:** 10.3389/fimmu.2019.01124

**Published:** 2019-05-22

**Authors:** Cordelia Manickam, Spandan V. Shah, Junsuke Nohara, Guido Ferrari, R. Keith Reeves

**Affiliations:** ^1^Center for Virology and Vaccine Research, Beth Israel Deaconess Medical Center, Harvard Medical School, Boston, MA, United States; ^2^Department of Surgery, Duke University School of Medicine, Durham, NC, United States; ^3^Ragon Institute of Massachusetts General Hospital, MIT, and Harvard, Cambridge, MA, United States

**Keywords:** HIV, SIV, non-human primates, innate immunity, natural killer cells, animal models

## Abstract

Natural killer (NK) cells are the major innate effectors primed to eliminate virus-infected and tumor or neoplastic cells. Recent studies also suggest nuances in phenotypic and functional characteristics among NK cell subsets may further permit execution of regulatory and adaptive roles. Animal models, particularly non-human primate (NHP) models, are critical for characterizing NK cell biology in disease and under homeostatic conditions. In HIV infection, NK cells mediate multiple antiviral functions via upregulation of activating receptors, inflammatory cytokine secretion, and antibody dependent cell cytotoxicity through antibody Fc-FcR interaction and others. However, HIV infection can also reciprocally modulate NK cells directly or indirectly, leading to impaired/ineffective NK cell responses. In this review, we will describe multiple aspects of NK cell biology in HIV/SIV infections and their association with viral control and disease progression, and how NHP models were critical in detailing each finding. Further, we will discuss the effect of NK cell depletion in SIV-infected NHP and the characteristics of newly described memory NK cells in NHP models and different mouse strains. Overall, we propose that the role of NK cells in controlling viral infections remains incompletely understood and that NHP models are indispensable in order to efficiently address these deficits.

## Introduction

**Figure 1:**
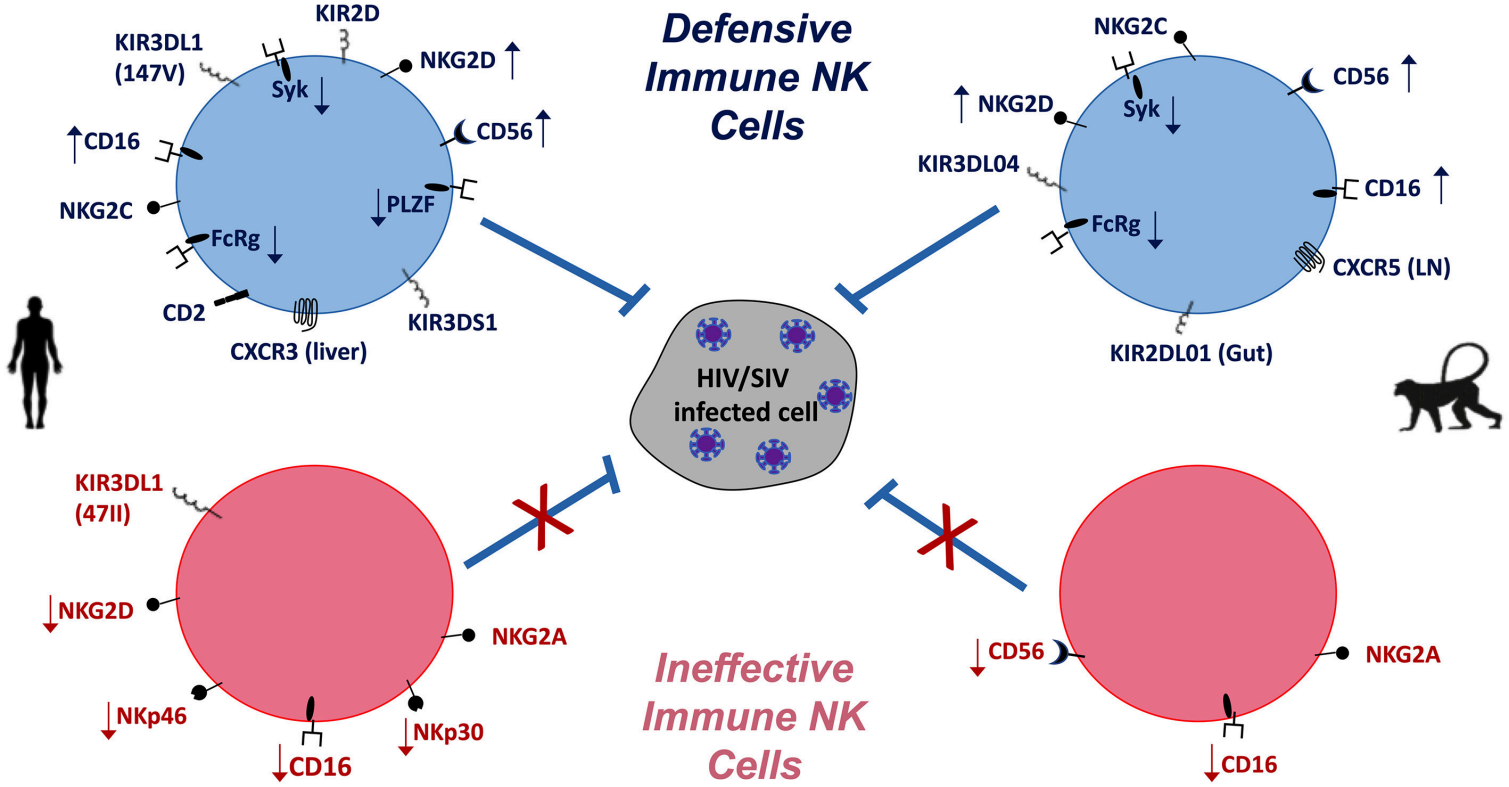
NK cells in HIV/SIV infections of humans **(Left)** and macaques **(Right)**. Differential expression (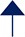 increase, 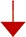 decrease) of NK cell markers indicative of defensive and effective anti-HIV/SIV functions (blue) are compared to virus-induced impaired (pink) functions. Overall, the balance of activating and inhibitory receptors is indicative of NK cell functionality and contribution to pathogenic outcomes.

Natural killer (NK) cells have previously been thought simplistically and aptly named, but recent characterizations suggest their roles in both innate and adaptive immunity are in fact quite diverse and complex. In humans, traditional phenotyping identifies NK cells as large non-B, non-T cells expressing CD56 and CD16, and in peripheral blood they are broadly classified into two subpopulations—CD56^bright^ cytokine-secreting and CD56^dim^CD16^+^ cytotoxic cells. The major function of NK cells in viral infections and cancer is lysis of target cells by rapidly releasing cytolytic mediators such as perforin and granzyme B and/or secretion of inflammatory cytokines which include but are not limited to interferon (IFN)-γ, tumor growth factor (TGF), tumor necrosis factor (TNF), interleukin (IL)-6, IL-10, granulocyte macrophage-colony stimulation factor (GM-CSF), and G-CSF. NK cell functions are controlled by a balance of activating receptors such as natural cytotoxicity receptors (1) (NKp30, NKp44, and NKp46), activating killer immunoglobulin receptors (KIRs) and C-type lectin receptors (NKG2D and NKG2C), and inhibitory receptors including inhibitory KIRs and NKG2A (2, 3). Recent studies in humans and mouse models have uncovered the existence of an array of NK cell subsets of diverse phenotypes and differential functions. Indeed, NK cell diversity in a single individual could range from 6,000 to 30,000 distinct phenotypes (4) and their functional repertoire now includes long lived memory-like responses, antigen specific memory responses and immunoregulatory roles in addition to their previously known innate functions (5–13). Given their unique nuances in phenotype, maturation, and function in blood and different tissue compartments, it is imperative to understand the role of NK cells in infections, specifically mucosal infections such as human immunodeficiency virus (HIV) and others. To this end, animal models, both mice and non-human primates (NHP), have proved useful in deepening our knowledge on NK cell biology, subsets, and tissue specific responses in health and disease. In the context of animal models to recapitulate the role of NK against HIV-1 infection, this review will primarily focus on these aspects of NK cells in human responses and its analogous modeling in non-human primates (NHP), which has been summarized in Figure 1.

## Caveats of Modeling Human NK Cell Biology in Mice

Mouse models have played significant and historic roles in understanding the interplay of the immune system and infections, with basic NK cell biology as a particularly notable example. However, critical differences between human and murine NK cells can sometimes complicate direct comparisons. Murine NK cells do not express CD56 but have approximate functional homologs—CD11b^low^CD27^high^ and CD11b^high^CD27^low^ NK cells have been correlated to the human CD56^bright^ and CD56^dim^ subsets respectively (14). However, the CD27^low^ subset is not capable of antibody dependent cell cytotoxicity functions (ADCC). Murine NK cells lack NKp44 and NKp30 expression altogether, and indeed NKp46 is the only NCR that is expressed on both murine and human NK cells (15). Further, while both murine and human NK cells express NKG2D, the ligands differ between the species. Murine NKG2D binds to 3 members of the minor histocompatibility family, 5 members of the retinoic acid early inducible gene 1 (Rae-1) family of proteins and murine UL-16-binding protein-like transcript (MULT1). Human NKG2D ligands include MHC I-like (MIC) molecules, MHC-I chain-related A, MHC-I chain-related B and UL16 binding (ULBP) protein family (16). The MIC family proteins are highly polymorphic with more than 70 alleles, and the NKG2D ligands of both species diversified independently, and thus are not orthologous (17). The trafficking markers, and hence the tissue distribution, also vary between the two species. For example, human NK cells are generally present homeostatically in lymph nodes (LN), albeit at low levels, whereas NK cells are observed in murine LN only after stimulation (18). A major difference, as well as an evolutionary disparity, is the recognition of their cognate MHC class I molecules. Murine NK cells use the Ly49 family of proteins which have C-type lectin domains, for cognition of MHC I molecules (19). Human NK cells lack Ly49 proteins and rather express the highly divergent Ig superfamily receptors called KIR that recognize MHC-I (20). Both Ly49 and KIRs act as functional analogs, but they vary to a large extent in their genetic and structural properties and exhibit qualitative differences in their MHC-I interaction. While mouse studies have significantly expanded our current knowledge of NK cell subsets and their functionality, some of these significant differences complicate the modeling of NK cell biology for some human diseases. Indeed, the limited lifespan, differences in antibody repertoires, and most critically for HIV-1 infection, the lack of lentiviral tropism in mice, has restricted their use as an animal model to understand the role of NK cells against HIV-1.

## Modeling NK Cell Biology in NHP

NHP NK cells are generally much more similar to human NK cells than murine NK cells and the possibility of *in-vivo* manipulations, such as depleting NK cell numbers, offer opportunities to specifically address NK cell biology. The peripheral NK cell frequency in Old World monkeys, which includes rhesus, cynomolgus and pig-tailed macaques, sooty mangabeys and African green monkeys (AGM), averages ~10% of blood lymphocytes similar to humans. Whereas, in neotropical primates such as common marmosets and cotton-top tamarins, the NK frequency is typically < 5% (21–26). Phylogenetic studies comparing multiple mammalian species have identified KIR3DL as the first ancestral gene originating from simian primates (27). Similar to human NK cells, great apes and Old World monkeys have a rich diversity of KIR3DL1, whereas the New World monkey KIRs diverged from the Old World monkeys, apes and humans, and their KIR3DL1 is more specific to their species. NHP NK cells also have a few dissimilarities such as the low expression of CD56, universal expression of CD8α and NKG2A/C by all subsets of NK cells compared to human NK cells (21, 25, 26, 28, 29). Due to this, the major delineating markers commonly used to identify NK cells in Old World and New World monkeys are CD8α/NKG2A/C and NKp46 respectively.

NHP NK cells, particularly those in rhesus macaques (MAC), have been studied in detail over the last two decades. Gating for CD56 and CD16 expression on circulating NKG2A/C^+^ MAC NK cells, defines three distinct populations: CD56^+^CD16^−^ cells which are functionally equivalent to human CD56^bright^ NK cells; CD56^−^CD16^+^ cells corresponding to the human CD56^dim^ NK cells and the CD56^−^CD16^−^ (DN) cells for which an analogous phenotype in humans is not yet clearly defined (30, 31). Although NK cell differentiation is dynamic, the CD56 expression pattern can denote the functional maturation of human NK cells, whereby downregulation of CD56 expression indicates a mature differentiated cytotoxic profile (32–34). Hong et al. (35) identified expression patterns in MAC NK cell subsets similar to human NK cells by transcriptional analysis. Expression pattern of transcripts in MAC CD56^+^ cells were consistent with primitively differentiated cytokine producing cells evidenced as IL-7R, TNF receptor super family member 1B, GATA-3, TCF-7, CD53, amphiregulin, and Granzyme K among others. Conversely, transcripts of effector proteins, such as CCL3, CCL4, and CCL5, were highly expressed in CD16^+^ cells. Interestingly, Hong et al. (35) found the DN subset to be an intermediary stage between the CD56^+^ and CD16^+^ subsets based on the transcriptional profile. While CD57 has also been proposed as a marker of mature, functionally distinct population of NK cells in humans (36), a simian analog has not been identified yet. Overall, the phenotypic, functional and transcriptional profiling has shown that NHP NK cells are well-suited to model their human counterparts as it will be discussed in the following sections.

## NK Cell Modulation of HIV and SIV Infections Via KIR/HLA

Epidemiological studies of long-term non-progressors and elite controllers of HIV infection have indicated that the co-expression of KIR3DS1 and a specific HLA-B haplotype known as the HLA-Bw480I correlates with lower viral load, a slower decline of CD4^+^ T-cell counts and delayed progression to AIDS (37–39). In fact, the NK cell subsets upregulate KIRs and KIR-like molecules in their effort to control virus replication as demonstrated by the protective role of HLA-Bw480I that can potentially bind KIR3DL1 on the membrane of NK cells, contribute to their expansion (40) and increase their cytolytic function (41). In addition to the polymorphism in the HLA-Bw4 variants associated with protection from disease progression, it has been recently reported that a single isoleucine-to-valine substitution in position 47 (I47V) of the KIR3DL1 was responsible for a less protective role in controlling HIV-1 infection compared to the 47VV (not reaching significance) and a significantly more protective role than the 47II genotype (42); the protective role was confined to its interaction with the HLA-B^*^57:01 and not with the HLA-B^*^57:03. These data suggest that the KIR-HLA interaction is specifically tuned to impact control of HIV-1 replication. These observations are also supported by the findings that both KIRDL2 and KIRDL3 expressing NK cells can mediate control of HIV-1 via interaction with HLA-C molecules (43, 44). In addition to the polymorphism of the KIR receptor, higher copy numbers of KIR3DS1 and KIR3DL1 in the presence of their ligands were associated with lower viral set point. NK cells from individuals with multiple copies of KIR3DL1 in the presence of KIR3DS1 and their ligands were able to inhibit *in vitro* replication more robustly (37).

Similar observations on the importance of the interaction between KIR and class I HLA molecules have been reported in MAC models and linked with their ability to control SIV infection. The polymorphisms of MAC KIR differs from human (45), but several activating KIR, defined as KIR3DL or KIR3DH, have been associated with lower virus load and longer survival alone or in association with the class I Mamu-A1^*^001 allele (46) [see also review by Walter and Ansari FI 2015 (47)]. Enhanced copies of KIR3DL04 (or KIR3DH04) were also reported to associate with decreased loss of CD4^+^ T cells and increased CD56/16 DN NK production of IFN-γ (48). Lastly, a unique profile of circulation and tissue accumulation of the KIR3DL01^+^ NK subset during acute SIV infection was reported to indicate that this subset (and not the KIR3DL05^+^) accumulate in gut tissues. These cells also displayed higher proliferation, activation, and antiviral function during chronic infection (49). The study did not correlate the findings with the outcome on control of viremia or disease progression, but raises the important issue of the dynamic changes that take place within the NK subsets during infection, with regard to their frequencies and tissue distribution. An important outcome for the recognition of infected cells by NK cells through the KIR/HLA interaction is related to the ability of NK cells to exert immune pressure on HIV-1 sequence (50, 51), similar to what was described for the CD8^+^ T cell responses (52, 53).

As discussed above, KIRs play important roles in controlling both HIV and SIV infections. However, one must be judicious when using NHP models to study the impact of KIRs on retroviral infection, due to the disparities between the KIR repertoires of human and that of NHP (54). For instance, the KIR subtype with only one extracellular domain, KIR1D, has been identified in MAC, which seems to be unique to NHP and there is no corresponding counterpart in human (55). Even though the nucleic acid sequence contains two Ig-like domains, the expressed protein is truncated at the second domain due to a frame shift mutation (55). Consequently, the KIR1D subtype lacks the cytoplasmic domain as well, and although it's *in vivo* function is still currently unknown, it is speculated to be secreted extracellularly (56). Another major feature that differentiates the human and NHP KIR is their complexity of KIR2D and KIR3D subtypes. Based on the structure and MHC molecule specificity, the KIR genes can be divided into 4 main lineages, I, II, III, and V (54, 57). While only 3 distinct lineage II KIR3D genes have been characterized in human, MAC has been reported to have a highly diverse lineage II repertoire, consisting of 10 KIR3DL genes and 9 KIR3DS genes (56, 58, 59). This may have evolved to complement the expanded HLA-A and B genes that are observed in NHP, and indeed, some of the KIR3DL/S has been shown to bind to HLA-A/B molecules (27, 58, 60). Conversely, humans have more diverse lineage III KIR genes that are absent in Old-World monkeys, such as MAC (55, 61), which is consistent with the higher variability of HLA-C molecules in human (62). Thus, the KIR gene repertoire for NHP seems to be much more complex than that of humans. The latest studies identified a total of 22 different KIR genes for MAC (59, 63), as compared to 15 KIR genes and 2 pseudogenes in humans (64). In general, the complexity of KIR genotype and expression in relation to the class I HLA molecules should be carefully considered when expression of KIR on human and NHP NK subsets are evaluated for their correlation with the outcome of retroviral infections. Furthermore, it is clear that, in both humans and NHP, KIR, and HLA class I polymorphism can influence the outcome of infection, but the evolutionary advantage of these molecules has not been elucidated yet.

## Fc-Receptor (FcR) Mediated NK Cell Functions in HIV/SIV Infections

Among the immunomodulatory and effector functions mediated by NK cells, their role as effector cells in Fc-dependent antibody functions represented by ADCC is very important in HIV-1 and SIV infections. This is best highlighted by the correlation between vaccine-induced ADCC responses and control of virus replication (65–68) and protection (69), and in pre-clinical studies conducted in MAC and recent observations in mother-to-child transmission (70, 71). Moreover, the only human vaccine clinical trial that provided limited success, the RV144 study conducted in Thailand, suggested a crucial role for non-neutralizing Ab responses capable of mediating ADCC as correlates with lower risk of infection (72, 73). The NK cells provide the effector cell component to these type of responses, upon engagement of their FcγR III (CD16) by the Fc region of an antibody that can recognize antigens expressed on the membrane of infected cells (74). In humans as well as in NHP, the canonical ADCC-mediating effector NK cell subset has been described as those that are lineage negative (lacking expression of markers defining major T and B cell subsets) and CD16^+^, which are the peripheral CD56^dim^CD16^bright^ cells in humans and CD3^−^CD20^−^CD8^+^NKG2A/C^+^CD16^+^in MAC. The effector function of these cellular subsets is in general regulated by the fine interaction between the Ab subclasses and the polymorphisms of the FcRs that present substantial differences between the human and NHP (75–77). In addition to the classical Fc-FcR engagement of ADCC effector cell subsets, it has also been reported that recognition of the infected target cells requires engagement of NKG2D-receptor, suggesting that the NKG2D may serve as a co-receptor for ADCC-mediated NK cell functions (78). In addition to the CD8α^+^CD16^+^ NK subset, it has been reported that the CD8α^−^ NK cells can also be potent ADCC effector cells in MAC and co-express the CD56, CD16, NKG2D, and KIR2D receptors. These cells represent approximately 35% of the macaque CD8α^−^ cells and are responsive to stimulation by IL-15 to upregulate the CD69 receptor and produce IFN-γ and TNF- cytokines, providing additional functions to the cytotoxicity (79).

## Impact of HIV and SIV Infection on NK Distribution and Function

In healthy humans, tissue NK cells are more heterogeneous, complex and less studied than their peripheral blood counterparts due to limited access to human tissues. Tissue-resident NK cells differ by their pattern of chemokine and adhesion receptors, which are specialized based on their homing properties and/or *in-situ* maturation (80, 81). CD56^bright^ NK cells in human blood express trafficking markers CD62L, CCR7, CXCR3, and CXCR4 that allow their migration into secondary lymphoid organs, inflamed tissues, and tumors, whereas tissue resident CD56^bright^ NK cells do not express CD62L but other adhesion markers such as CD49a and CD103 (18, 82–84). The CD56^dim^ subset expresses receptors that are necessary for migration into inflamed sites including CXCR4, CX3CR1, CXCR2, and CXCR3 and low levels of CD62L and no CCR7. On the other hand, CD56^bright^ NK cells express high levels of CCR7 and CD62L and constitute a large proportion of NK cells in the lymph node because of their affinity to high endothelial venules (HEV) (18, 85). In fact, LNs have been proposed as a site of maturation for some NK cells (86). CD56^bright^ NK cells are also the predominant population in the gut and participate in the gut homeostasis (87). However, multiple pathogens including HIV-1, can disrupt the overall homeostatic NK cell distribution in tissues.

It has been previously reported that NK cells undergo redistribution amongst different tissue compartments during the acute phase of HIV infection, as indicated by the increased frequency of circulating CD3^neg^CD56^neg^CD16^pos^ NK cells with perturbed functional profiles and reduced presence of CD3^neg^CD56^pos^ NK cells (88). Despite this body of evidence that suggests an important role for NK cells in the control of HIV-1 replication, they are not able to clear the infection. This may be attributed, at least in part, to the overall subversion of the immune system caused by HIV-1, where NK cells are not only altered functionally but may be impaired in trafficking and tissue infiltration. In fact, it was described very early that NK cells were dysfunctional in HIV-1 infected subjects (89) and this effect could be detected at the level of their ability to perform FcR-mediated functions (90), as well as expression of KIR, activation markers, and cytokine production (91, 92). The impairment of NK cells could be due to the direct effect of HIV-1 or, due to the effect of cytokine milieu on NKp30 and NKG2D expression (93) and/or CD4 dysfunction. The latter has been recently demonstrated to be the case because blockade of PD-1 and IL-10 pathways can restore the HIV-1 specific CD4 T cells *in vitro* and enhance cytokine expression and cytolytic function of the NK subsets (94). The presence of impaired NK subsets is not solely observed during HIV-1 infection as reported by Meier et al. (95). In fact, they described a similar alteration of the NK subsets in HIV-1 and Hepatitis C virus (HCV) infections with a clear decline in the frequency of the CD56^dim^ NK that resulted in reduction of IFN-γ production and cytotoxic function (95). Similar observations on the impairment of NK cell function has been described in SIV-infected MAC related to differentiation, cytokine secretion, and expression of activation/homing markers (96, 97). The importance of these dysfunctions in the context of hampering the ability of NK subsets to fully control retroviral infection is indicated by the demonstration that in chronically SIV-infected MAC, the frequency of CD56/16 DN NK cells in the spleen and liver of infected animals with high virus load was significantly lower than in animals with lower virus load (98). Moreover, frequency of the liver-resident CXCR3^+^ NK cells and circulating NKG2D^+^ cells were inversely correlated with plasma viremia (98). These data suggest that differences in the location and function of the NK subsets have a relevant impact on the outcome of virus replication in the SIV model and the same may occur in HIV-1 infection. To support the complexity of this reality, data was collected from the animals that can naturally survive the SIV infection. In fact, the analysis of the distribution of NK cells within the LN in the pathogenic and non-pathogenic SIV-infection models represented by the MAC and AGM, respectively, revealed unique differential aspects (99). In the pathogenic model, the NK cells were found in a random distribution and did not accumulate in the follicles, whereas a significantly higher frequency of NK cells was observed in the AGM LN mostly around or within the follicles. The AGM NK cells also expressed CXCR5 and the frequency of CXCR5^+^ NK cells in the AGM LN was significantly higher. This distribution persisted throughout the time of observation of the infected animals and was associated with a significantly higher frequency of cells with membrane-bound IL-15 in the AGM. Anti-IL-15 treatment of AGM depleted NK cells from LN, spleen, and gut, and it induced a significantly increased plasma viral load as well as the amount of cell-associated viral RNA and DNA in the LN, compared to the untreated animals. Collectively, these data indicated that the unique control of virus in non-pathogenic AGM is at least partially mediated by NK cells.

## Impact of *in vivo* NK Depletion in SIV-Infected Animals

A major argument for the importance of CD8^+^ T cell responses in control of retroviral infection was initially provided by seminal studies conducted by Letvin and collaborators, who reported the immediate rebound in SIV replication in MAC upon depletion of CD8^+^ T cells by infusion of targeted monoclonal antibodies (100). Similar experiments have now been conducted in MAC models to address the role of NK cells in virus control, but thus far have provided contrasting results. Initially, CD16^+^ NK depletion performed using the 3G8 mAb 24 h before infection with SIV did not impact the level of viremia observed in the infused animals during the first 11 days of infection compared to those receiving a control mAb. These data suggested that CD16^+^ NK cells did not contribute to initial control of SIV replication (101), although the assay had several caveats, such as the emergence of idiotypic antibodies (102) and the lack of CD16^−^NK cells depletion from lymph nodes that are largely responsible for controlling virus replication (85, 103). More recently, depletion of NK cells in the periphery and in intestinal mucosal tissues following administration of JAK3 inhibitors induced a modest but significant increase of plasma viral load in all six animals tested and in tissue viral load in 5 out of 6 animals. The latter was not related to an increase in frequency of CD4^+^ T cells, suggesting an increased production of the virus on a per cell basis. (104). A follow-up study investigated prolonged administration of the JAK3 inhibitor during the acute phase of infection and recapitulated the finding of significant higher virus replication during the chronic phase (>12 weeks) of infection in JAK3-treated MAC, but not during the acute phase of infection. One caveat to the latter study was related to the concomitantly observed partial depletion of CD8^+^ T cell subsets, among other immune cells, and the unresolved contribution that this could have had on the outcome of the study, mainly implicating the depletion of NK subsets in the gastrointestinal tissue (105). The different outcomes of these studies could either be related to the stage of infection, acute vs. chronic infection, or to the depletion procedure, that could impact different NK cell subsets. Overall, the data do not provide a definitive determination of the impact that NK cells could have on the control of SIV infection in MAC. The anti-IL-15 neutralization approach to deplete NK cells has been shown to be effective in AGM and MAC (99, 106), but full evaluation has not been performed during acute and chronic infection of a pathogenic species.

## Memory NK Cells and HIV/SIV

The possibility of NK cells with adaptive features perhaps emerged from an unexpected observation by Boehncke et al. (107) wherein wild-type (WT) and T-cell deficient mice responded similarly to 4-dinitro-1-fluorobenzene (DNFB)-induced contact hypersensitivity (CHS). NK cell memory, as an emerging field of study, was further solidified by O' Leary et al. (108) in a CHS mouse model with the observation of T- and B-cell independent adaptive immunity that was mediated by NK cells. These responses were elicited by haptens and persisted for at least 4 weeks following sensitization. The same group later demonstrated (109) that liver-resident NK cells in mice were not only capable of generating a memory pool against haptens but also against influenza, vesicular stomatitis virus (VSV) and HIV; and that the chemokine receptor CXCR6 plays a critical role in this process. Subsequently, the phenomenon has since been observed in other mouse models, non-human primates, as well as in humans (reviewed in (12, 110, 111)).

As our understanding of the memory NK cell response expands, multiple subpopulations that may mediate antigen recall through differing mechanisms have emerged. These different subtypes are, however, somewhat fluid. For practical purposes, we will comment on four categorizations of these cells:

1. True *antigen-specific memory NK* cells respond to an antigen presented analogously to classical adaptive cells. Antigens include haptens (112), cytomegalovirus (CMV) (13, 113–115), HIV (13, 109), and others (109, 116, 117).
2. *Cytokine-induced memory* NK cells seem to respond to specific cytokines (IL-12, IL-15, and IL-18) with a brief pre-activation period followed by enhanced activity in response to cytokine receptor stimulation (118). Cytokine-induced memory NK cells have been reported against influenza virus (119), leukemia (120) and melanoma (121). A recent article also reports the generation of “tumor-induced memory-like” (TIML-) NK cells (122).
3. *Memory-like* (adaptive) NK cells comprise a unique subset of NK cells that have reduced expression of the CD16 adaptor molecules, FcR γ-chain, and Syk, specifically induced in responses to CMV infection (123–127). Memory-like NK cell numbers have also been shown to expand in HIV (128, 129), HCV (130) and Epstein Bar Virus (EBV) (131–133) infections.
4. *Evolved memory* (adaptive) NK cells overlap with antigen-specific and memory-like NK cells with a response induced by CMV infection. A critical difference is the expression of a specific receptor, Ly49H, by NK cells that interact with MCMV glycoprotein m157 (134, 135) in mouse models, and more recently, Ly49I and Ly49C were also shown to interact with specific MCMV peptides (136). An analogous NKG2C^+^ cell type has also been described in humans although the mechanisms are less well-defined (137). Collectively, an evolved memory NK cell can be considered as one that expresses a receptor (or perhaps a precise combination of known and unknown receptors) that is induced to control a specific pathogen.

Paust et al. (109) demonstrated that murine NK cells can develop memory against HIV antigens, a virus which cannot infect mice, and thus there is no evolutionary component. Primed hepatic NK cells (but not splenic NK cells) mounted a vigorous recall response in recipient mice, and the chemokine receptor CXCR6 was deemed critical for this function. Later, our group showed that NK cells in MAC were capable of mounting a recall response against SIV/SHIV and HIV vaccine antigens (13), and NKG2C was indicated to play a critical role in this process.

To our knowledge, memory-like FcR γ-chain deficient NK cells have not been reported in mice. One possibility is the exclusive association of CD16 to FcR γ-chain homodimers in mice, compared to the association with homodimers and heterodimers of FcR γ-chain and CD3ζ in humans (138). Thus, murine NK cells might not be able to respond to CMV infection in a manner similar to human NK cells. Nonetheless, the prevalence of other subtypes of NK cells (cytokine-induced and antigen-specific) suggests murine NK cells have likely devised alternate strategies to control viral infections. Other such subtle variations of these cells almost certainly exist, but the exact interplay between the pathogen and NK cells that induces each population still needs significant assessment.

The defining characteristics of “memory-like” NK cells are the lack of the FcR γ signaling chain and Syk adaptor proteins, likely resulting from epigenetic reprogramming of these subsets of NK cells by CMV (126, 139). The initial observation made by Leeansyah et al. suggested persistent lack of FcR γ-chain expression in NK cells from HIV-1 positive subjects receiving cART (140); however, CMV infection status of these patients was not reported in this study. A follow-up study by the same group suggested that these cells had significantly reduced NCR (NKp46 and NKp30) expression and showed greater ADCC against opsonized targets (128), but the role of HIV *per se* in the induction of these cells is not clear. CMV is likely the primary source of induction of these cells (124, 125), but how HIV can also modulate these phenotypes in NK cells is unclear. Our own assessment in MAC suggests SIV infection does not have a significant impact on total numbers of γ-chain^−^Syk^−^ NK cells, which is modulated by rhesus CMV (rhCMV), but the migration into tissues was heavily influenced by SIV infection (127). In blood, rhCMV titers were correlated to adaptive NK cell numbers in both rhCMV-infected, as well as rhCMV/SIV co-infected animals (127). This observation was similar to observations made by Zhou et al. (128), where adaptive NK cell prevalence generally correlated with CMV antibody titers, but was further modulated by HIV infection. This discrepancy could simply be explained by host species-specific differences, or perhaps by HIV specific responses exerted by NK cells in humans. Furthermore, SIV was able to subvert the enhanced responses of adaptive NK cells by suppressing the alternate signaling mechanism induced by rhCMV (127). The data explaining the effect of HIV/SIV infection on adaptive NK cells, in the absence of prior CMV infection, are sorely lacking. It is imperative to address the skewed observations in SIV infection, which could have arisen due to the confounding effects of co-infection with CMV. Most importantly, we need to address the question of protective features of CMV induced NK cells against other viral infections such as HIV-1.

Similar questions have been posited for other memory NK cell subsets as well, particularly NKG2C^+^ evolved NK cells induced by CMV infection (141). Similar to the expansion of γ-chain^−^Syk^−^ NK cell populations in SIV/HIV^+^ subjects, the expansion of the NKG2C^+^ population in HIV-1^+^ subjects is attributed to concurrent HCMV infection (142). A recent study suggests that adaptive NK cells induced by CMV in HIV-1 infected individuals are further modulated and marked by reduced expression of the transcription factor promyelocytic leukemia zinc finger (PLZF) (129, 129) and that these cells are distinguished from other adaptive NK cells expressing NKG2C or CD57. Intriguingly, HIV might be directly affecting NKG2C^+^ NK cell numbers, since p24 has been reported to stabilize HLA-E expression on lymphocytes of HIV^+^ patients (143). Overall, the questions regarding the protective ability of memory/adaptive/cytokine-induced NK cell subsets against HIV remain unanswered. Information on the unadulterated effect of HIV on the NK cell receptor repertoire, functional abilities, epigenetic reprogramming and specific subset expansion *in vivo* needs significant in-depth investigation and may direct specific preventative and curative strategies against HIV infections.

## Concluding Remarks

Many studies have highlighted the crucial role of NK cells in mediating control of HIV transmission, dissemination, disease, and reciprocally virus-mediated subversion of NK cells. Unlike many other pathogens, mouse models have contributed in a more limited way to this body of knowledge, due to the lack of tropism of lentiviruses and caveats of humanized mouse models. These circumstances have created one of the best examples of the significant utility of studying immunology in NHP, specifically SIV infection of various MAC species. Overall, these studies have revealed multiple layers of NK cell-virus interplay in lentivirus infection including: (1) KIR-HLA; (2) induction of CD2 and NKG2-related molecules; (3) interaction of Fc-receptor bearing NK cells and Ab-opsonized virus infected cells; and (4) development of HIV/SIV-specific NK cell memory-like responses. Although many unanswered questions remain regarding NK cell correlates of virus control, the significant contribution of NHP models cannot be overstated and rapidly evolving *in vivo* and *ex vivo* manipulations will undoubtedly continue to advance studies of HIV vaccine and other therapeutic modality development.

## Author Contributions

CM, SS, JN, and GF contributed to writing of specific sections. RR and GF oversaw overall preparation of the manuscript, contributed to writing. RR edited the final version of the manuscript.

### Conflict of Interest Statement

The authors declare that the research was conducted in the absence of any commercial or financial relationships that could be construed as a potential conflict of interest.
